# Piperine, a Bioactive Component of Pepper Spice Exerts Therapeutic Effects on Androgen Dependent and Androgen Independent Prostate Cancer Cells

**DOI:** 10.1371/journal.pone.0065889

**Published:** 2013-06-18

**Authors:** Abhilash Samykutty, Aditya Vittal Shetty, Gajalakshmi Dakshinamoorthy, Mary Margaret Bartik, Gary Leon Johnson, Brian Webb, Guoxing Zheng, Aoshuang Chen, Ramaswamy Kalyanasundaram, Gnanasekar Munirathinam

**Affiliations:** 1 Department of Biomedical Sciences, University of Illinois, College of Medicine, Rockford, Illinois, United States of America; 2 Midwest SciTech, Bloomington, Minnesota, United States of America; 3 Thermo Fisher Scientific, Rockford, Illinois, United States of America; Van Andel Institute, United States of America

## Abstract

Prostate cancer is the most common solid malignancy in men, with 32,000 deaths annually. Piperine, a major alkaloid constituent of black pepper, has previously been reported to have anti-cancer activity in variety of cancer cell lines. The effect of piperine against prostate cancer is not currently known. Therefore, in this study, we investigated the anti-tumor mechanisms of piperine on androgen dependent and androgen independent prostate cancer cells. Here, we show that piperine inhibited the proliferation of LNCaP, PC-3, 22RV1 and DU-145 prostate cancer cells in a dose dependent manner. Furthermore, Annexin-V staining demonstrated that piperine treatment induced apoptosis in hormone dependent prostate cancer cells (LNCaP). Using global caspase activation assay, we show that piperine-induced apoptosis resulted in caspase activation in LNCaP and PC-3 cells. Further studies revealed that piperine treatment resulted in the activation of caspase-3 and cleavage of PARP-1 proteins in LNCaP, PC-3 and DU-145 prostate cancer cells. Piperine treatment also disrupted androgen receptor (AR) expression in LNCaP prostate cancer cells. Our evaluations further show that there is a significant reduction of Prostate Specific Antigen (PSA) levels following piperine treatment in LNCaP cells. NF-kB and STAT-3 transcription factors have previously been shown to play a role in angiogenesis and invasion of prostate cancer cells. Interestingly, treatment of LNCaP, PC-3 and DU-145 prostate cancer cells with piperine resulted in reduced expression of phosphorylated STAT-3 and Nuclear factor-κB (NF-kB) transcription factors. These results correlated with the results of Boyden chamber assay, wherein piperine treatment reduced the cell migration of LNCaP and PC-3 cells. Finally, we show that piperine treatment significantly reduced the androgen dependent and androgen independent tumor growth in nude mice model xenotransplanted with prostate cancer cells. Taken together, these results support further investigation of piperine as a potential therapeutic agent in the treatment of prostate cancer.

## Introduction

Western men are confronted with an increasing incidence of cancer and cancer related deaths annually. Statistics indicate that prostate cancer is the second leading cause of cancer related deaths among the men in United States. According to the recent estimates in the United States, 217,730 men will be newly diagnosed with prostate cancer and 32,050 men will die of this disease in 2010 [1]. Prostate cancer initially begins as being hormone dependent but as the disease progresses it transitions into being hormone independent and resistant to hormone related treatment. Currently available treatment options such as chemotherapy, radiotherapy, surgery or hormonal therapy are unsatisfactory [2]. Natural products, derived from plants or microorganisms, have become a key source of anti-cancer therapies, with a substantial number of current therapies being either natural or derived from natural products. Therefore, there is a great deal of interest in identifying natural compounds in the treatment of prostate cancer. Evidence is accumulating that compounds of plant origin (phytochemical) exert anti-cancer effects with less toxicity [3]. Black pepper, the spice of the millennia has been widely used in various food preparations throughout the globe. In the United States alone, the average daily intake of black pepper has been estimated at 359 mg. Piperine accounts for 5% to 9% of the black pepper content, implying the daily intake of approximately 60–110 µM [4]. Piperine (trans-trans isomer of 1-piperoyl piperidine) is the active principle and the main ingredient of black pepper used as a traditional medicine in India [5]. The potential of piperine as anti-cancer agent has been demonstrated previously. Piperine inhibited solid tumor development in mice induced with DLA (Dalton Lympoma Ascites) cells and extended the life span of mice bearing Ehrlich ascites tumor [6]. Piperine has also been shown to have anti-invasion activity of B16F-10 melanoma cells [7]. The cytoprotective effect of piperine on B (α)-p (Benzopyrene) induced experimental lung cancer has been successfully investigated in mice and inferred that piperine could exert its chemopreventive effect by modulating lipid peroxidation and augmenting antioxidant defense system [8]. Interestingly, recent studies have demonstrated that piperine can inhibit breast cancer by targeting the cancer stem cell renewal properties [9].

Despite its wide use and its ability to inhibit several cancer types, little is known about the beneficial effects of piperine against prostate cancer. Makhov and colleagues [4] previously showed that co-administration of docetaxel and piperine resulted in enhanced anti-tumor efficacy in a xenograft model of human castration-resistant prostate cancer via inhibition of CYP3A4 activity. To date, however, no other studies have characterized the direct anticancer effects of piperine in prostate cancer cells despite being shown to enhance the chemotherapeutic potential of docetaxel against prostate tumors [4]. Therefore, the objective of the study is to determine the anti-prostate cancer activities of piperine, as well as to determine the underlying molecular mechanisms of its action.

## Materials and Methods

### Ethics Statement

Animal experiments was performed in this study according to the guidelines set for the care and use of laboratory animals and with the rules formulated under the Animal Welfare Act by the United States Department of Agriculture (USDA). The protocol was approved by the IACUC Committee of the University of Illinois, College of Medicine at Rockford and animal studies performed at a facility accredited by AAALAC and USDA.

### Chemicals and reagents

Fetal calf serum (FCS), RPMI-1640 and Minimum Essential Medium (MEM) were obtained from American Type Cell Culture (ATCC), Manassas, VA,USA. Piperine was purchased from Sigma-Aldrich (St. Louis, MO). Cell viability assay kit was purchased from Dojindo Molecular Technologies Inc., Gaithersburg, MD. Annexin V-FITC apoptosis detection Kit was obtained from MBL international, Woburn, MA.

### Cell lines and cell culture

LNCaP, DU-145, 22RV1 and PC-3 cell lines were obtained from American Type Cell Culture (ATCC), Manassas, VA, USA and cells were cultured in RPMI medium supplemented with 10% FCS and 50 µg/mL gentamycin. For all experiments, 1×10^5^ cells/ml were seeded and grown for 24 h before experimental treatments. Cells were maintained at 37°C, 5% CO_2_ environment.

### Cell viability assay

LNCaP, 22RV1, DU-145 and PC-3 cells were seeded in 96-well tissue culture plates and incubated until cells attached to wells. All the cells were then treated and incubated with different concentrations of piperine (5–200 µM) for 24, 48 and 72 h. Cell viabilities were determined using a cell counting kit-8 (CCK-8) from Dojindo Molecular Technologies. 10 µL CCK-8 solution was added to the piperine treated cellsand incubated for 3 h. Optical density was measured at 450 nm using a BIO-RAD microplate reader model 680.

### Caspase activation assay

LNCaP and PC-3 cells were seeded in 96-well tissue culture plates and cultured until they reached 50% confluency. Prostate cancer cells were then treated and incubated with different concentrations of piperine (50–200 µM) for 24, 48 and 72 h respectively. Each plate was then incubated with 2 µL fluorescently-labeled caspase probe (NIR-FLIVO 747 In Vivo Apoptosis Tracer, Immunochemistry Technologies, LLC, Bloomington, MN) for 15 minutes. Cells were washed with 100 µL 1× PBS to remove caspase substrate. Following this, 100 µL 1× PBS was added to 24 h plate and 100 µL of the complete medium was added to the 48 h and 72 h plates so cells would not dry out before being read. Plates were read using a LI-COR Odyssey machine V3.0 to detect global caspase activation.

### Annexin V-FITC staining for apoptosis detection

LNCaP cells were cultured in an 8-chamber tissue culture slide in RPMI-1640 medium supplemented with 10% fetal bovine serum (FBS) and gentamycin until cells attached to wells. Cells were then treated and incubated with different concentrations of piperine (60 µM and 75 µM) for 24 hrs. Medium was then removed from wells and used later for the PSA assay. Apoptosis was determined using an Annexin V-FITC apoptosis detection kit from MBL International. Cells were stained by adding 500 µL of binding buffer, 5 µL Annexin, and 5 µL PI to each well and incubating in dark at room temperature for 5–10 minutes. Binding buffer, Annexin and PI was then removed from wells along with the chamber. 2–3 drops of 1× PBS was added to each section of the slide and covered. Slide was then analyzed using a fluorescence microscope.

### PSA assay

PSA assay was performed using the supernatants collected from LNCaP cells treated with piperine (5–150 µM). Prostate specific antigen secretion (ng/mL) was determined using a Human Prostate-Specific Antigen ELISA Kit purchased from Abnova.

### Western blot analysis

LNCaP and PC-3 cells treated with 60 µM and 75 µM of piperine respectively and DU145 cells treated with 160 µM for 24 h. Additionally, LNCaP cells were also treated with 25 µM of piperine to determine the low dose effects. Following treatment, cells were lysed with sample solubilizing buffer and subjected to SDS-PAGE, transferred to nitrocellulose membrane for Western blot analysis. Following antibodies were used for immunoblotting. Anti-NF-kB (MBL International Inc.), anti-caspase-3 (eBioscience), anti-PARP-1 (SantaCruz Biotechnology), anti-STAT-3 and anti-phospho STAT-3 (Cell signaling Technologies), anti-PSA (Thermo Fisher), anti-Androgen Receptor (Sigma Aldrich) and anti β-Actin-peroxidase(Sigma Aldrich) antibodies were used with vendor's recommended dilutions. Cells treated with 0.1% DMSO solvent served as controls.

### Boyden chamber assay

LNCaP and PC-3 cells were seeded in a Transwell® (Corning) chamber, treated and incubated with piperine concentrations of 60 µM and 75 µM respectively for 24 h. Cells were then removed from the top of the membrane using a pipette and any remaining cells were removed using a Q-tip. A HEMA 3 staining set from Fisher Scientific was used to fix and stain the cells. Following this, each membrane was rinsed with water and any remaining stain was removed from the top of each membrane using a Q-tip. Membranes were analyzed for cell migration using a light microscope (Nikon).

### Animals

6 weeks old male nude mice weighing approximately 20 grams were maintained in the Animal Facility at the University of Illinois at Rockford College of Medicine. All experimental procedures using animals were approved by the Institutional Animal Care and Use Committee of the University of Illinois at Rockford College of Medicine. LNCaP (5×10^6^) and DU145 (1×10^6^) cells suspended in equal volume of matrigel were injected subcutaneously into the flank regions of the male nude mice and tumors were allowed to grow. Once the tumor reached 50 mm^3^ in size, mice were treated daily with piperine (100 mg/kg) homogenously prepared in vegetable oil by intraperitoneal injections for 1 month. Control group were injected with vegetable oil alone. The effects of piperine on prostate tumor growth in nude mice were also tested by oral gavage administration as described previously [10]. For gavage study, LNCaP cells (7×10^6^) suspendend in matrigel was subcutaneously implanted in nude mice. Following 24 hours after implantation of LNCaP cells, mice were treated daily with piperine (10 mg/kg body weight) prepared in PBS by oral gavage. Control animals received PBS alone gavage treatment. Following 1 month of treatment, mice were sacrificed by carbon dioxide inhalation followed by exsanguination and tumors were excised and measured the mass and volume. Tumor volumes were calculated by the formula: [Volume = 0.5×(Width)^2^×length. The above *in vivo*experiment was in concordance with ARRIVE guidelines [11].

### Statistical Analysis

Statistical analysis was performed with Graph Pad Prism 5 software. Data were compared using Student's t-test. P<0.05 was considered statistically significant.

## Results

### Piperine inhibits proliferation and induces death in both androgen dependent (AD) LNCaP and androgen independent (AI) DU145, 22RV1, PC-3 cells in vitro

**Figure 1:**
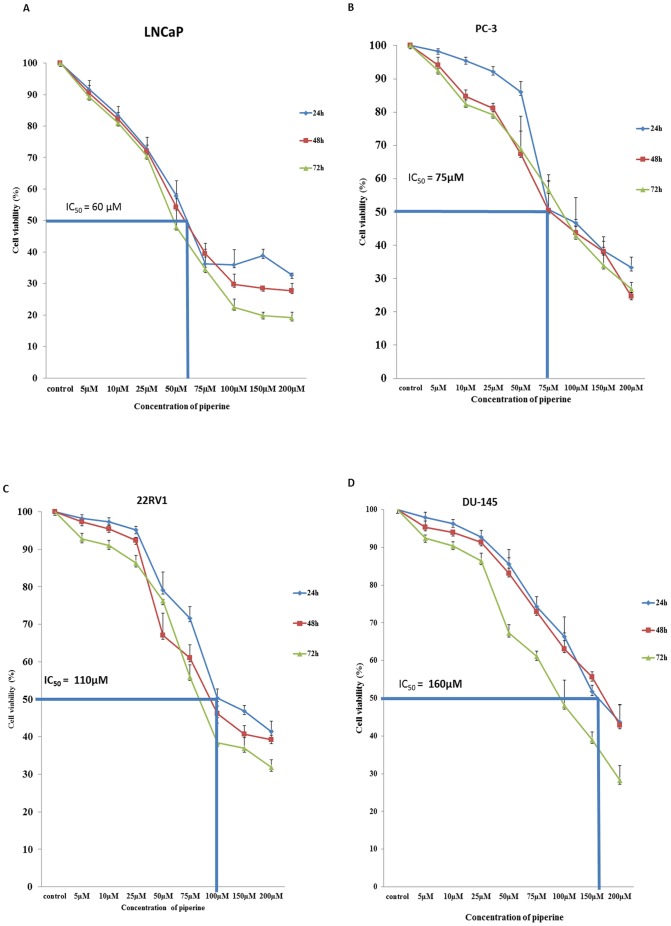
Piperine inhibits cell proliferation in androgen dependent and androgen independent prostate cancer cell lines. Piperine inhibits cell proliferation of LNCaP, PC-3, 22RV1 and DU-145 with an IC50 of about 60 µM, 75 µM, 110 µM and 160 µM in the respective prostate cancer cells. Results showed that piperine inhibited proliferation of both androgen dependent (LNCaP) and androgen independent derived prostate cancer cells (PC-3, 22Rv1 and DU145) in a time and dose dependent manner. Data presented is representative of one of three similar experiments.

We first determined the anti-proliferative effects of piperine on human prostate carcinoma cells including androgen sensitive (LNCaP) (Figure 1A) and androgen insensitive (PC-3, 22Rv1, DU-145 cells: Figure 1B–1D). The cells were treated with (5–200 µM) piperine for 24, 48, 72 hours. The treatment of LNCaP (AD) and PC-3 (AI) cells with piperine resulted in significant reduction of proliferation or viability in a dose dependent manner with an IC-50 values of 60 µM and 75 µM respectively, as assessed by MTT. In case of 22Rv1 and DU-145 prostate cancer cells, piperine treatment exhibited higher IC-50 values of 110 µM and 160 µM respectively. Thus, piperine seems to be capable of exerting a differential level of cytotoxic effects depending on the type of prostate cancer cells with androgen dependent prostate cancer cells (LNCaP) being the most sensitive one. The IC50 values obtained from this cell viability assay results were used to evaluate the effects of piperine on prostate cancer cells in subsequent experiments.

### Piperine treatment reduces Prostate Specific Antigen (PSA) levels in LNCaP cells

**Figure 2:**
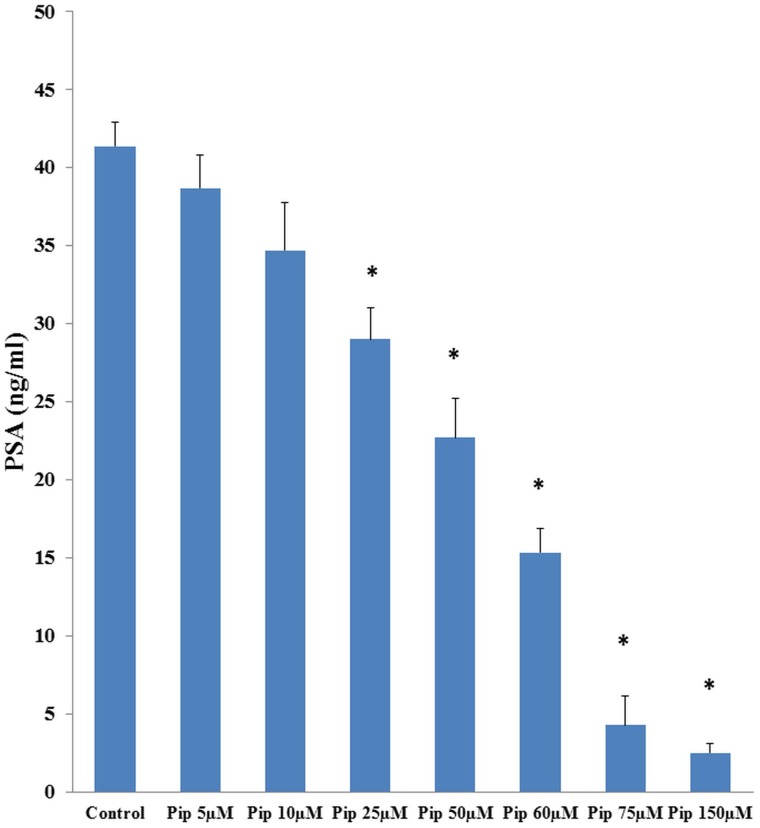
Piperine treatment down regulates PSA expression in LNCaP cells. PSA assay results showed that piperine has dose dependent effects on the secretion of PSA (downstream target of AR) in LNCaP cells. * p<0.05 compared to control LNCaP cells.

PSA is the gold standard marker used in diagnosis and monitoring treatment efficacy of prostate cancer. Our initial studies showed that AR-positive LNCaP cells were sensitive to piperine treatment. Piperine treatment at 75 µM concentration significantly inhibited the secretion of PSA to near normal level (4.244 ng/ml) compared to untreated LNCaP cells (41.24 ng/ml) (Figure 2). Interestingly, piperine at a low dose range of 25 µM to 60 µM also had a significant impact on PSA secretion from LNCaP cells.

### Piperine induces apoptosis in PCa cells: Annexin-V FITC staining analysis and caspase activation assay

**Figure 3:**
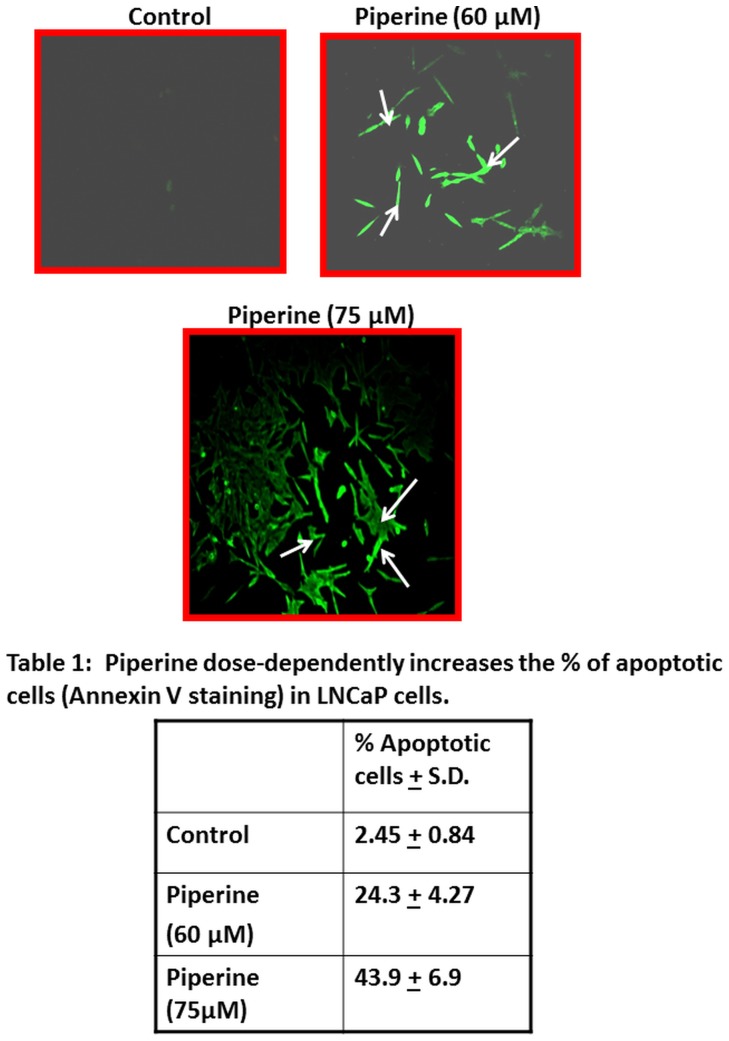
Piperine induces apoptosis in LNCaP cells. Annexin-V-FITC staining of the LNCaP cells shows that cells treated with piperine were positive for annexin V binding as evident from fluorescence signal. Arrow indicates cells positive for Annexin-V staining. Apoptotic cells were quantified based on the number of cells positive for Annexin-V staining compared to total cells present in each field. Results show that increasing the concentrations of piperine led to increased apoptosis as shown in the table 1 in Figure 3. Representative results of one of three independent evaluations.

To determine whether the reduction in proliferation and cell viability of prostate cancer cells by piperine was associated with the induction of apoptosis, LNCaP cells were treated with different concentrations of piperine and the number of apoptotic cells was assessed using the Annexin V– apoptotis detection kit as previously described [12]. LNCaP cells were treated with 60 µM or 75 µM of piperine for 24 h and stained with Annexin V-FITC and propidium iodide to visualize the cells under fluorescent microscope. Fluorescent microscopic analysis demonstrated that LNCaP cells treated with piperine resulted in an increased number of apoptotic cells compared to control LNCaP cells (Figure 3) in a dose-dependent manner (60 µM and 75 µM respectively). Based on the above results in which we determined the concentration of piperine on inhibition of cell proliferation and induction of apoptosis, we selected a piperine concentration of 60 µM for further mechanistic studies with the LNCaP cells.

**Figure 4:**
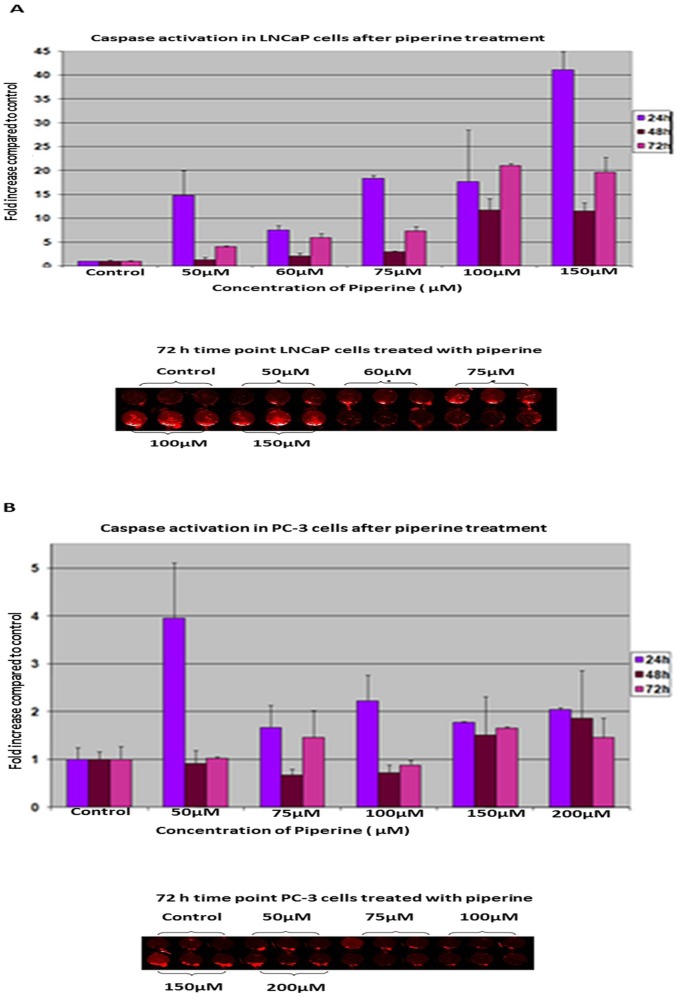
Piperine induced caspase activation in prostate cancer cell lines. A NIR-FLIVO 747-conjugated poly-caspase probe, which is a cell-permeable fluorescent detector of active caspases, was employed to determine whether piperine activates caspase in prostate cancer cells. The LNCaP and PC-3 cell lines were treated with piperine and then tested for caspase activation. Results showed that piperine induced global caspase activation in both LNCaP (A) and PC-3 cells (B) as early as 24 hours. Experiments were repeated four times and obtained similar results as shown in this representative figure.

In addition to Annexin-V-staining, we analyzed apoptosis in LNCaP and PC-3 cells by Global Caspase activation assay. Caspase is a valuable and reliable marker for apoptosis. We therefore analyzed its activation in LNCaP and PC-3 PCa cell lines after treatment with piperine by global caspase activation assay using fluorescently-labeled poly-caspase probe (Figure 4). The cells were incubated with 50–200 µM of piperine at different time points (24, 48, 72 hours). Both LNCaP and PC-3 cells treated with piperine resulted in increased caspase activation. The increase in caspase activation in LNCaP cells was in a dose and time dependent manner whereas PC-3 exhibited consistently high levels of caspase activation at both the concentration and at all the time points except at 48 hours, where caspase activation seemed to decrease and increase again. This may be due to the different sensitivities of cell during various time points. Taken together, these results indicate that piperine-induced apoptosis in both LNCaP and PC-3 cells via caspase activation.

### Western blotting analysis: Piperine treatment activates the expression of caspase-3 and cleaves PARP-1

**Figure 5:**
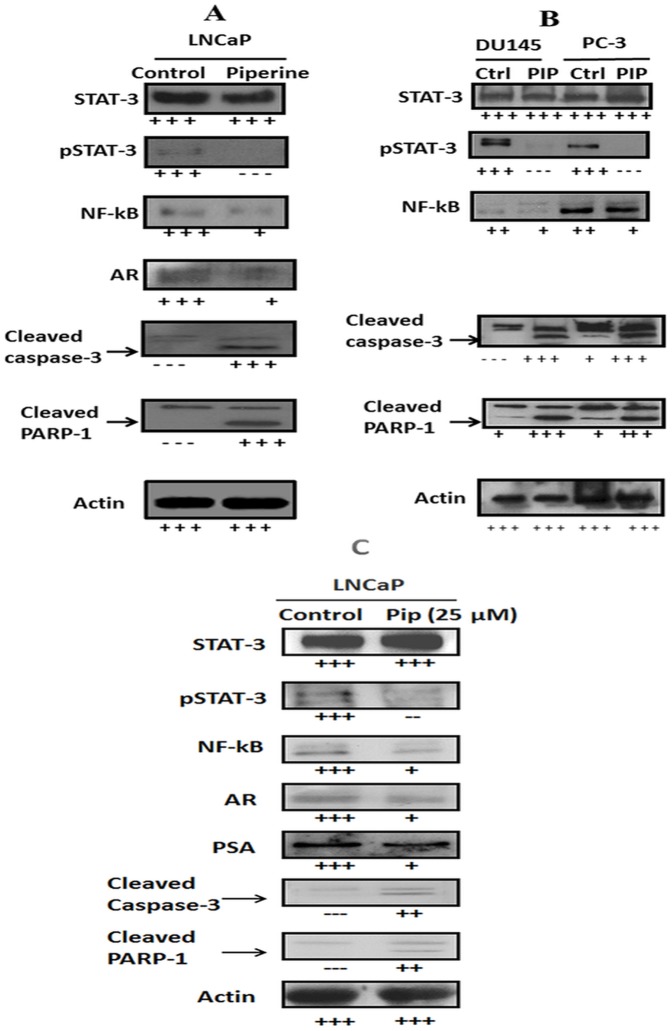
Piperine activates apoptotic markers in androgen dependent and androgen independent prostate cancer cells by targeting AR, NF-kB and STAT-3 transcription factors. (a) Western blot analysis showed that 60 µM piperine inhibits the expression of AR, STAT-3 and NF-kB transcription factors in LNCaP cells while simultaneously activating apoptotic signals (Caspase-3 and PARP-1 activation). (b). Western blot analysis showed that 160 µM and 75 µM piperine treatment inhibits the expression of STAT-3 and NF-kB transcription factors in DU-145 and PC-3 cells by activating apoptotic markers (caspase-3 and PARP-1 activation). C) Immunoblot results showed thatpiperine at lower dose of 25 µM also inhibits the expression of AR, STAT-3 and NF-kB transcription factors in LNCaP cells in addition to downregulating PSA expression. Changes in the expression of proteins are indicated by + or − sign. LNCaP, DU-145 and PC-3 cells treated with 0.1% DMSO alone served as controls (ctrl) in this experiment.

The activation of executioner caspases, i.e caspase-3, results in the cleavage of a broad spectrum of cellular target proteins, including poly (ADP-Ribose) polymerase-1 (PARP-1), leading to the cell death [13]. Therefore, we determined the effect of piperine on the activation of caspase-3 and Poly (ADP) Ribose Polymerase. Immunoblot analysis of LNCaP (Figure 5A and Figure 5C) and DU-145, PC-3 cells (Figure 5B) treated with piperine resulted in an increase in the cleavage of caspase-3 and PARP-1 when compared with cells treated with DMSO alone. These results were in concordance with the global caspase activation that we observed before (Figure 4).

### Piperine treatment down regulates the expression of Androgen Receptor (AR), NF-kB and phosphorylated STAT-3

NF-kB and STAT-3 (phosphorylated form of STAT-3) transcription factors and Androgen Receptor (AR) play a critical role in cell proliferation, anti-apoptosis, angiogenesis and invasion of prostate cancer cells. Immunoblot analysis of LNCaP (Figure 5A) cells treated with 60 µM of piperine showed reduction in the expression of NF-kB and STAT-3 (phosphorylated form of STAT-3) transcription factors and downregulation of Androgen Receptor (AR) in these cells. Interestingly, lower concentration (25 µM) of piperine treatment also decreased the expression of phosphorylated STAT-3, NF-kB and PSA levels in LNCaP cells (Figure 5C). Our results also showed that DU-145 and PC-3 PCa (Figure 5B) cells treated with 160 µM and 75 µM of piperine dose respectively also resulted in the downregulation of NF-kB and phosphorylated STAT-3 expression levels, underscoring the anti-cancer effects of piperine in prostate cancer cells.

### Piperine treatment reduces cell migration in vitro

**Figure 6:**
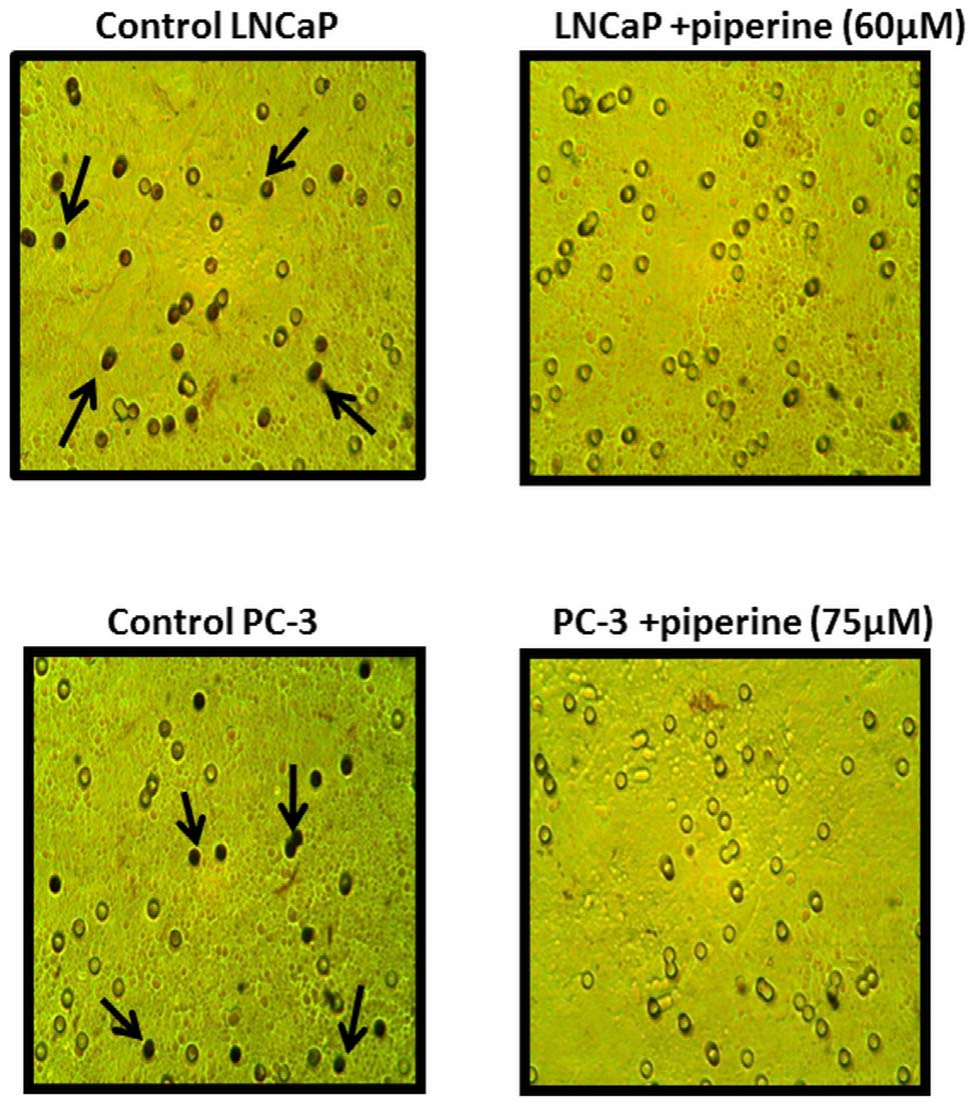
Piperine treatment abrogates migration of prostate cancer cells *in vitro*. Boyden chamber assay shows that control LNCaP and PC-3 prostate cancer cells have a greater number of migrated cells while LNCaP and PC-3 samples treated with 60 µM and 75 µM piperine show fewer migrated cells in Transwell® chambers. Arrow indicates migrated cells. The inhibition of cell migration suggests that piperine may have anti-migratory properties in prostate cancer. Data shown here is representative of one of three similar results obtained.

Piperine treatment reduced the cell migration of LNCaP and PC-3 cells, suggesting that piperine has anti-migratory effects in prostate cancer (Figure 6).

### Piperine administration inhibits tumor growth of human prostate cancer cell xenografts implanted in immunodeficient mice

**Figure 7:**
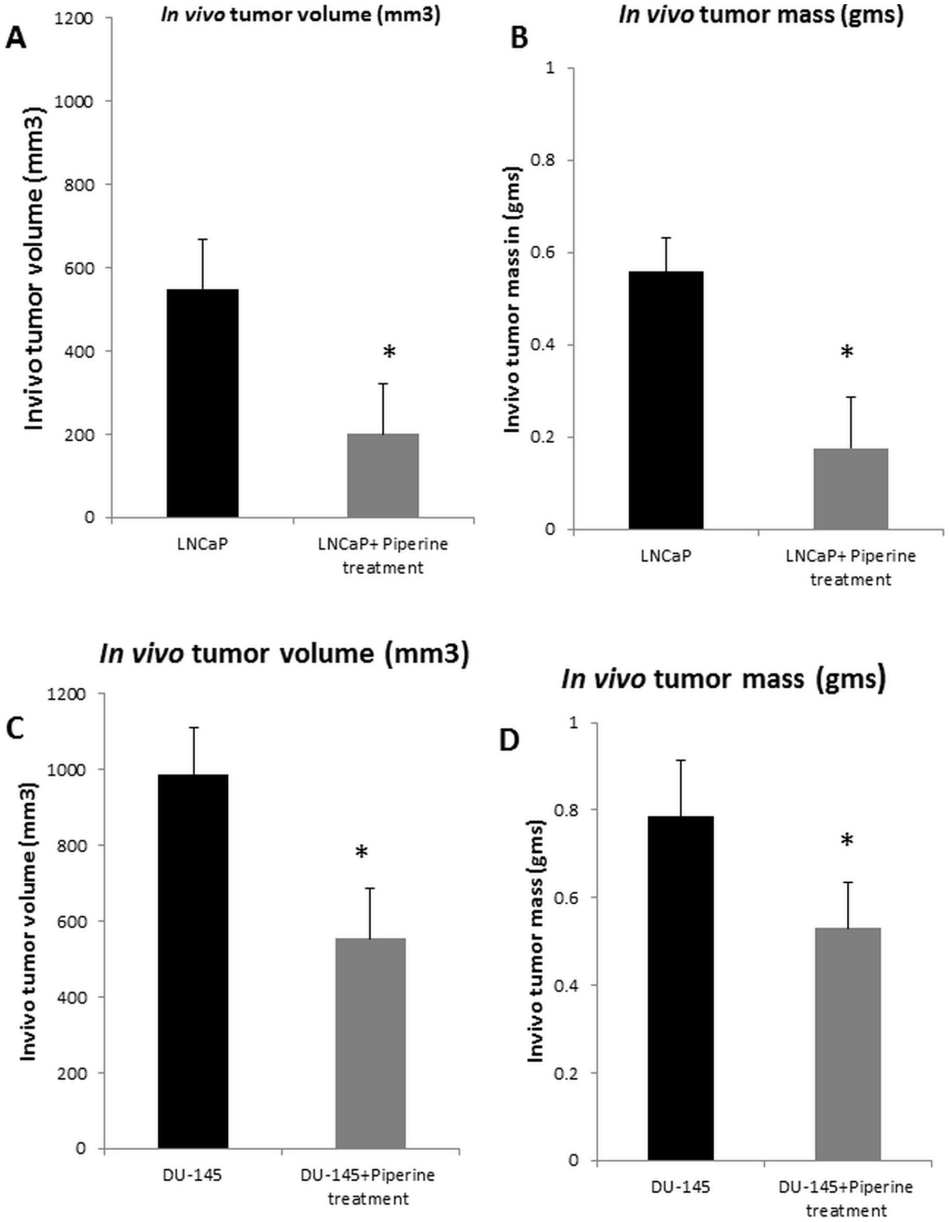
Effects of piperine on the growth of LNCaP and DU-145 derived xenografts in nude mice. Piperine inhibits the growth of LNCaP and DU-145 derived tumor xenografts in nude mice model. Tumor volume (mm^3^) and weights (gms) of the piperine treated and control untreated nude mice were measured on the indicated days. Six independent tumors were collected from the piperine treated LNCaP, DU-145 and control nude mice respectively. Results (A–D) showed that piperine injection significantly reduced the tumor volumes and tumor weight of both androgen dependent and androgen independent derived prostate cancer cells implanted in nude mice. *p<0.05 compared to control group.

**Figure 8:**
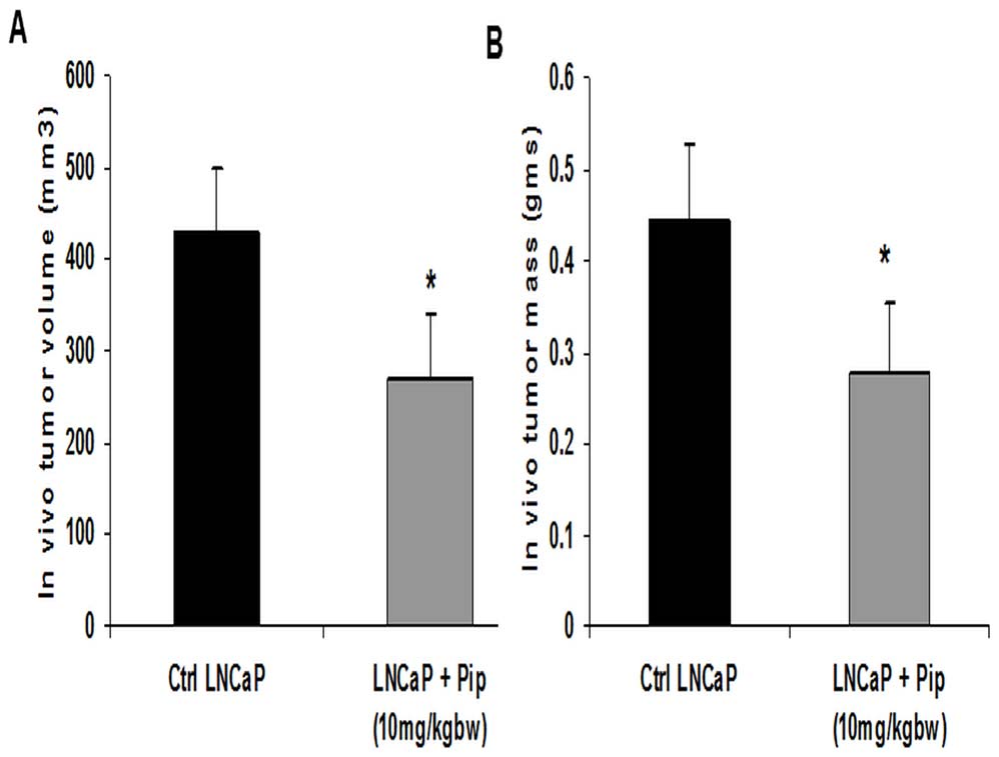
Oral administration of piperine inhibits LNCaP derived tumor growth in nude mice. Piperine given to nude mice (n = 6) at a dose of 10 mg/kg via gavage administration significantly inhibits the xenograft growth of LNCaP tumors compared to nude mice (n = 6) given PBS alone control group. After completion of treatment, tumors collected from nude mice were measured for tumor volumes and tumor weight. Results (A&B) showed that piperine significantly reduced both tumor volumes and tumor weight of LNCaP derived prostate cancer cells in nude mice. *p<0.05 compared to control group.

We next sought to determine the antitumor effects of piperine *in vivo* using a xenograft model in nude mice. As evident from the results, treatment with piperine significantly reduced tumor growth in nude mice implanted with LNCaP cells by 72% [tumor volume (p<0.01) and tumor mass (p<0.01)] (Figure 7A & 7B) and treatment of piperine also reduced the tumor growth in nude mice implanted with DU-145 cells by 41% [tumor volume (p<0.05) and tumor mass (p<0.05)] (Figure 7C & 7D). Importantly, piperine (10 mg/kg) administered to nude mice by gavage treatment also inhibited the tumor growth of LNCaP cells by 38% [tumor volume (p<0.05) and tumor mass (p<0.05)] (Figure 8A & 8B).The reduction in tumor mass and volume in piperine treated groups were significant. Based upon these results, piperine appears to have tumor suppressive effects on both androgen dependent and androgen independent prostate cancer cells *in vivo*.

## Discussion

Recent estimates in the sex ratio revealed the fact that the number of males between the ages 15 and 65 were tremendously increased which had a direct impact on the number of prostate cancer patients [14]. Various physical and genetically associated factors contribute to the progression of prostate cancer. The hope of prostate cancer treatment by “natural” dietary products also known as phytochemicals has become an active area of research. Piperine is one such promising compound abundantly present in pepper spice [15]. Thus far, there is no study which assesses the direct therapeutic effect of piperine on prostate cancer and only few studies have demonstrated its potential in other cancers [16],[17],[18],[19],[20]. Hence in the present study, we sought to evaluate the efficacy of piperine as anti-cancer agent against both androgen dependent and independent prostate cancer cell lines and investigate the molecular mechanisms responsible for its anti-proliferative activities. The anti-cancer effect of piperine was recently demonstrated in colon cancer cells [21]. In that study, piperine displayed a trend towards anti-proliferation on colon cancer cells at 24 hours and a significant level of inhibition on cell proliferation was reported at 48 and 72 hours respectively [21]. In another study, piperine was shown to effectively inhibit Benzo (α) pyrene induced lung carcinogenesis in albino mice by protecting proteins from damage and also by suppressing cell proliferation [8]. In the present study, we observed an anti-proliferative activity exhibited by piperine on androgen dependent (AD) LNCaP and androgen independent (AI) PC-3, DU-145 and 22RV1 PCa cells *in vitro* in a dose dependent manner. Results of our study revealed that LNCaP cells were more sensitive to piperine treatment followed by PC-3, 22RV1 and DU-145 cells suggesting that piperine acts differentially depending on the type of prostate cancer cell line.

The robust anti proliferative effect exerted by piperine on both androgen dependent and androgen independent prostate cancer cells can be considered advantageous in the treatment of prostate cancer. The early-stage prostate cancer depends on androgens for growth and survival, and androgen ablation therapy causes them to regress [22]. Cancers that are not cured by hormonal therapy eventually become androgen independent, rendering anti-androgen therapy ineffective [22],[23]. Furthermore, our investigations also revealed that piperine is an inducer of apoptosis in prostate cancer cells as evident from Annexin-V immunofluorescence staining studies.

The potential of piperine to promote apoptosis is further supported by its ability to enhance caspase activation in both androgen-dependent and androgen-independent prostate cancer cells. An effective therapeutic agent should not only limit proliferation but should also be capable of activating programmed cell death in both AD and AI prostate cancer cells [24], [25]. Given the fact that piperine effectively inhibited the proliferation of prostate cancer cells and induced apoptosis, piperine may be a promising anti-prostate cancer agent that merits further investigation as a chemopreventive or chemotherapeutic agent. The caspases, implicated in apoptosis, in general are classified as initiators and executioners of cell death [26]. In this study we found that there was a rapid increase in the global caspase activity elicited by piperine in LNCaP prostate cancer cells. Caspase-3 is the critical death related enzyme which executes apoptosis in prostate cancer cells [27]. Results presented in this study confirm that piperine treatment activated caspase-3 in prostate cancer cells. This was further confirmed in our studies as we observed cleavage of PARP-1 [28] in piperine treated prostate cancer cells. To further delineate the molecular mechanism by which piperine induces apoptosis, STAT-3 a key anti-apopoptic survival factor in prostate cancer cells was evaluated. STAT-3 plays an important role in cancer cell proliferation, migration and invasion in many types of cancer such as bladder, ovarian, and brain [29], [30], [31]. In prostate cancer, STAT-3 activation has been found to be associated with lymph node and bone metastases [32]. Among the STAT family of transcription proteins, inhibition of phosphorylated STAT-3 has been identified as a critical target for cell death by enhanced apoptosis in PCa cells [33],[34],[35],[36]. Hence, in the present study it can be postulated that piperine can induce apoptosis by inhibiting the activation of phosphorylated STAT-3 in LNCaP, DU145 and PC-3 cells which, in turn, may inhibit the survival and growth of prostate cancer cells.

In addition to inhibiting STAT-3, our results showed that piperine also targeted the expression of nuclear factor-kB (NF-kB) transcription factor and androgen receptor (AR). NF-kB plays important roles in the control of cell growth, differentiation, apoptosis and metastases [37],[38], [39]. The existing evidence from this present study also points that many anti-cancer effects of piperine may also be regulated either directly or indirectly by NF-kB. Our results suggest that NF-kB is down regulated in LNCaP, PC-3 and DU-145 PCa cells. Similarly androgen receptor (AR) was down regulated in LNCaP cells. The down regulation of AR was in consistent with the simultaneous reduction in the PSA expression in LNCaP cells treated with piperine. The fact that androgen dependent prostate cancer cells are more sensitive to piperine treatment could be due to down regulation of AR which is critical for the survival of androgen dependent prostate cancer cells such as LNCaP cells that we used in this study. Previous studies have shown that prostate cancer progression during androgen deprivation therapy was well correlated with the overexpression of NF-kB [40],[41],[42],[43]. In PCa cells, NF-kB can regulate the expression of AR. Androgen deprivation- resistant prostate cancer (ADRPC) express higher levels of AR transcript and protein. Resistance during prostate cancer therapy is mainly due to the marked increase in AR expression [44],[45],[46]. However, DU-145 and PC-3 cells do not express AR suggesting that AR independent survival mechanisms contributing in these cell types. Our results have shown that in LNCaP (AD) cells, piperine can down regulate both NF-kB and AR. Similarly, piperine reduced the expression of NF-kB in DU-145 and PC-3 (AI) cells. These results indicate that piperine could inhibit PCa of both AD and AI PCa cells. However, androgen dependent prostate cancer cells (LNCaP) were found to be more sensitive to piperine treatment due to downregulation of multiple targets such as AR, NF-kB and STAT-3 expression in these cells.

Our results presented in this study also suggest that piperine could inhibit migration abilities of LNCaP and PC-3 prostate cancer cells which may be beneficial in preventing metastases. This notion is backed by the Boyden chamber assay, where inhibition of cell migration of LNCaP and PC-3 prostate cancer cells is observed following treatment with piperine. In support of our findings, previous studies have shown that piperine also has an inhibitory effect on invasion of B16-F10 melanoma cells via NF-kB inhibition [7].

The anti-proliferative effect of piperine was also confirmed in our *in vivo* studies, where tumor growth was substantially reduced following piperine treatment in nude mice subcutaneously implanted with LNCaP and DU-145 cells. Based on our *in vitro* cell proliferation studies, we have selected the highly sensitive LNCaP and the least sensitive DU-145 cells for the comparison of efficacy of piperine treatment. Our results here show that piperine treatment (100 mg/kg body weight) via intraperitoneal injection against LNCaP xenotransplants resulted in a 72% reduction in tumor size as compared to the DU-145 treated group, where the reduction in tumor size was noted to be 41%. Interestingly, piperine at lower dose (10 mg/kg) administered via gavage treatment also inhibited LNCaP xenograft tumor growth by 38%. Hence, our *in vivo* results in nude mice correlated with our *in vitro* studies of piperine treated LNCaP and DU-145 cells. Previous studies have shown that piperine treatment on PC-3 prostate cancer cells *in vivo* resulted in a non-statistically significant reduction in tumor size [4]. We, therefore, did not select PC-3 cells for our *in vivo* studies. Our *in vitro* data, however, suggests that piperine has an antiproliferative effect on PC-3 cells. Makhov *et al* treated mice only twice, once a week, with piperine [4] but did not observe any significant reduction in tumor growth. In our study, mice in the treatment group were treated daily with piperine for 4 weeks. The significant reduction in tumor growth that we observed may be due to the different treatment regimen (dose and daily injections) used in our *in vivo* studies. Moreover, the piperine concentration especially the gavage administration (10 mg/kg body weight) used in our study seems to be tolerable in humans based on the following dose extrapolation studies. We have made an attempt to derive the human equivalent dose (HED) based on our *in vivo* piperine concentration used in treating the mice. Recent studies advocate the use of body surface area (BSA) as a factor when converting a dose for translation from animals to humans, especially for phase I and phase II clinical trials [47]. Based on BSA, the daily HED of piperine (10 mg/kg) is 48.6 mg/day, a dose which is closer to previously studied tolerable piperine dose (50 mg/day) in humans [48]. However, extensive studies are needed to determine the optimal tolerable dose of piperine in pre-clinical studies before advancing to human trials.

Taken together, our findings suggest that caspase-3 activation, PARP-1 cleavage, down-regulation of phosphorylated STAT-3, inhibition of NF-kB expression and AR may represent the molecular mechanism by which piperine disrupts cell proliferation and induces apoptosis especially in androgen dependent prostate cancer cells. Based upon the results presented here, further studies are clearly warranted to evaluate the therapeutic potential of dietary feeding of piperine against prostate cancer in experimental animal models.
